# The 4D Repair Technique for Incidental Durotomy in Spine Surgery: Surgical Technique and Clinical Outcomes in 112 Patients

**DOI:** 10.3390/jcm15218278

**Published:** 2026-10-24

**Authors:** Andrea Franchini, Giuseppe Rovere, Felice Barletta, Franco Lucio Gorgoglione, Andrea Perna

**Affiliations:** 1 Department of Orthopedics and Trauma Surgery, Fondazione Casa Sollievo della Sofferenza IRCCS, 71013 San Giovanni Rotondo, Italy; 2 Department of Clinical Science and Translational Medicine, Section of Orthopaedics and Traumatology, University of Rome Tor Vergata, 00133 Rome, Italy

**Keywords:** incidental durotomy, dural repair, cerebrospinal fluid leak, spine surgery, fibrin sealant, autologous graft, subfascial drainage

## Abstract

**Background:** Incidental durotomy is a recognized complication of spinal surgery and can cause persistent cerebrospinal fluid (CSF) leakage, wound complications, and reoperation. We describe the 4D repair technique and report postoperative outcomes from a single-center clinical series. **Methods:** This retrospective descriptive study included 112 patients treated between 2005 and January 2025. The 4D technique combines double-layer continuous dural suturing, fibrin sealant, an autologous dermal-fat graft, and passive subfascial drainage. Clinical characteristics and postoperative outcomes were summarized descriptively without a historical comparator. **Results:** Mean age was 64.1 ± 9.2 years; 60 patients (53.6%) were male. Persistent CSF leakage occurred in 3 patients (2.7%), symptomatic pseudomeningocele in none, CSF fistula in 1 (0.9%), and wound dehiscence in 2 (1.8%). Nine patients (8.0%) underwent reoperation for any indication. No postoperative meningitis was recorded. Mean hospital stay was 9.1 ± 3.8 days and mean passive drainage duration was 7.5 ± 2.3 days. **Conclusions:** This illustrated 112-patient experience describes the application and observed postoperative course of a standardized multimodal dural repair technique. The descriptive design does not establish comparative effectiveness or the independent contribution of individual steps.

## 1. Introduction

Incidental durotomy is a clinically important complication of spinal surgery, with reported frequencies that vary according to the operative indication, surgical complexity, and revision status [1,2,3,4,5]. Inadequate dural closure can lead to persistent CSF leakage, pseudomeningocele, wound disruption, headache, infection, and unplanned reoperation [4,5,6]. Multivariate and registry-based investigations have also examined risk factors and outcomes associated with incidental durotomy [7,8]. Management strategies include direct microsurgical suturing, dural substitutes or autologous grafting, fibrin or other sealants, and selective postoperative CSF diversion [9,10,11,12,13]. However, there is no universal agreement concerning the optimal combination of repair materials and postoperative care measures [9,10].

A standardized approach may facilitate reproducibility when dural tears occur in complex operative settings. Our institution introduced a four-component protocol, termed the 4D repair technique, integrating mechanical dural closure, sealant augmentation, biological reinforcement, and controlled passive drainage. The present work aims to provide a detailed illustrated description of that technique and to report clinical characteristics and outcomes among 112 treated patients. Published experience with alternative approaches is discussed for context; no historical control group is used for efficacy comparisons.

## 2. Materials and Methods

### 2.1. Study Design and Population

This retrospective, single-center descriptive case series included 112 adult patients with incidental intraoperative durotomy treated using the institutional 4D protocol from 2005 through January 2025. Patients undergoing spinal surgery for eligible degenerative or traumatic pathology were included when an incidental dural tear and perioperative records were documented. Patients with spinal infection, neoplastic disease, congenital malformation, intradural pathology, or incomplete records were excluded. Patients were identified through the institutional surgical registry. The analysis was confined to the 112 patients receiving the 4D technique; no earlier historical control cohort was included.

The study used routinely collected, anonymized clinical data. Informed consent for the scientific use of anonymized clinical data was obtained, as reported in the institutional source records. The retrospective analysis involved no additional study-specific intervention, and institutional policy did not require separate formal ethics committee approval. Reporting was guided by the STROBE statement [14].

### 2.2. The 4D Repair Technique

**Figure 1:**
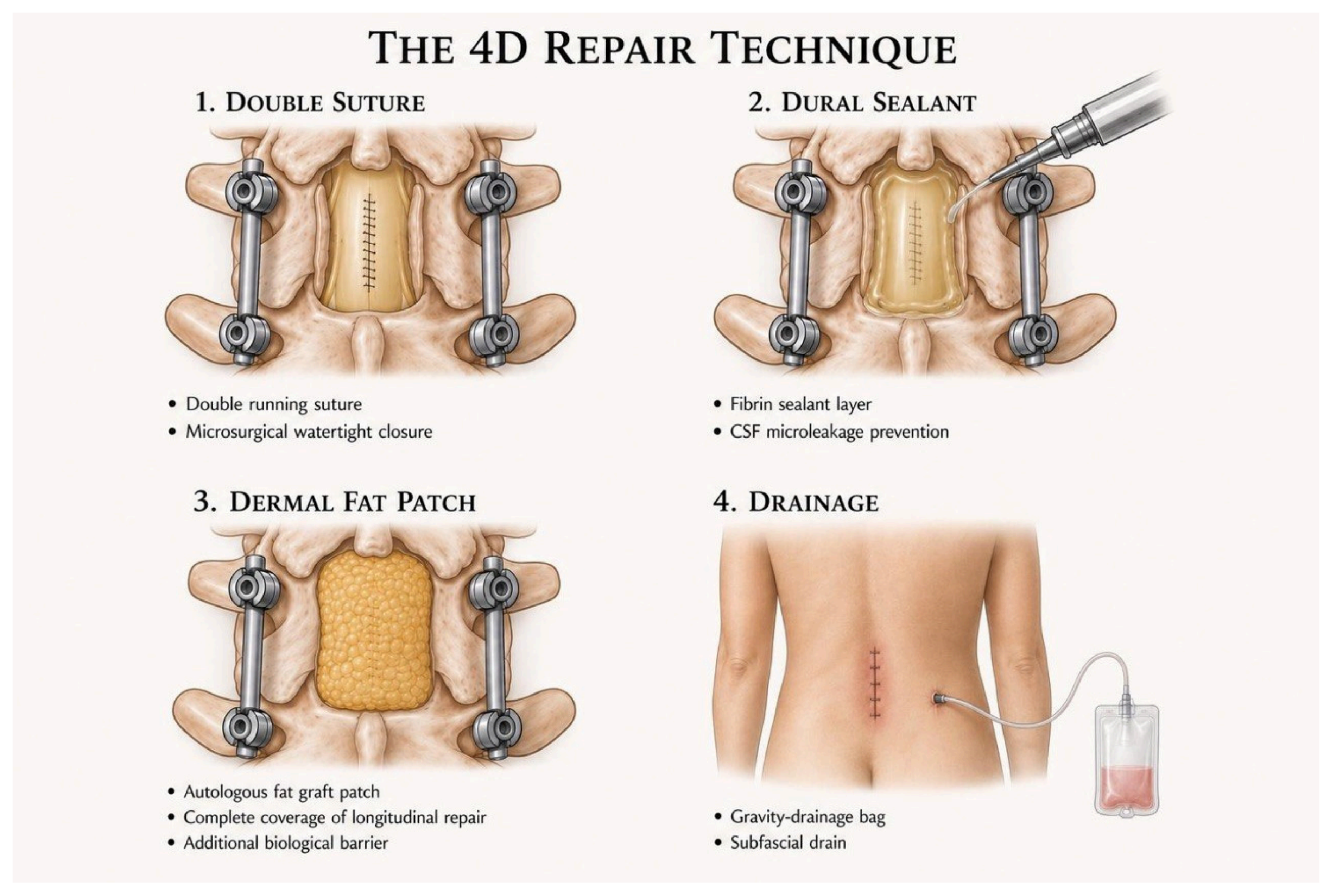
Schematic of the four 4D components: double dural suturing, fibrin sealant, autologous dermal-fat graft, and passive subfascial drainage.

The 4D technique is a sequential multimodal repair bundle, performed under microscopic magnification and combined with multilayer wound closure (Figure 1). The individual components are described below.

First D (double-layer dural closure): Primary watertight closure was achieved under high magnification using a double continuous running suture with 6-0 Prolene (Ethicon, Somerville, NJ, USA). The double-layer configuration was designed to optimize circumferential load distribution and reduce focal stress concentration at the dural edge, thereby improving resistance to early postoperative CSF pulsatility.

Second D (dural sealing reinforcement): A standardized layer of fibrin sealant (Tisseel^®^, Baxter International Inc., Deerfield, IL, USA) was applied over the suture line to enhance immediate sealing integrity and reduce microleakage during the early healing phase.

Third D (dermal-fat biological reinforcement): An autologous dermal-fat graft was positioned over the repaired dural segment as an additional biological covering. In revision cases, graft material could be harvested through the same incision from available soft tissue and scar-associated tissue, avoiding a separate donor-site incision. As the graft is not described as pedicled, it should be regarded as a free autologous graft rather than a vascularized flap.

Fourth D (directed low-pressure drainage): A passive 2.2-mm subfascial drain was placed adjacent to the repair site and maintained without active suction. The system was designed not as a therapeutic variable but as an integral component of a controlled pressure-regulation strategy, aimed at reducing dead-space accumulation while avoiding the negative-pressure gradient associated with CSF siphoning. Drain removal was performed after a minimum of 7 days and only following mobilization once output was <50 mL/24 h.

**Figure 2:**
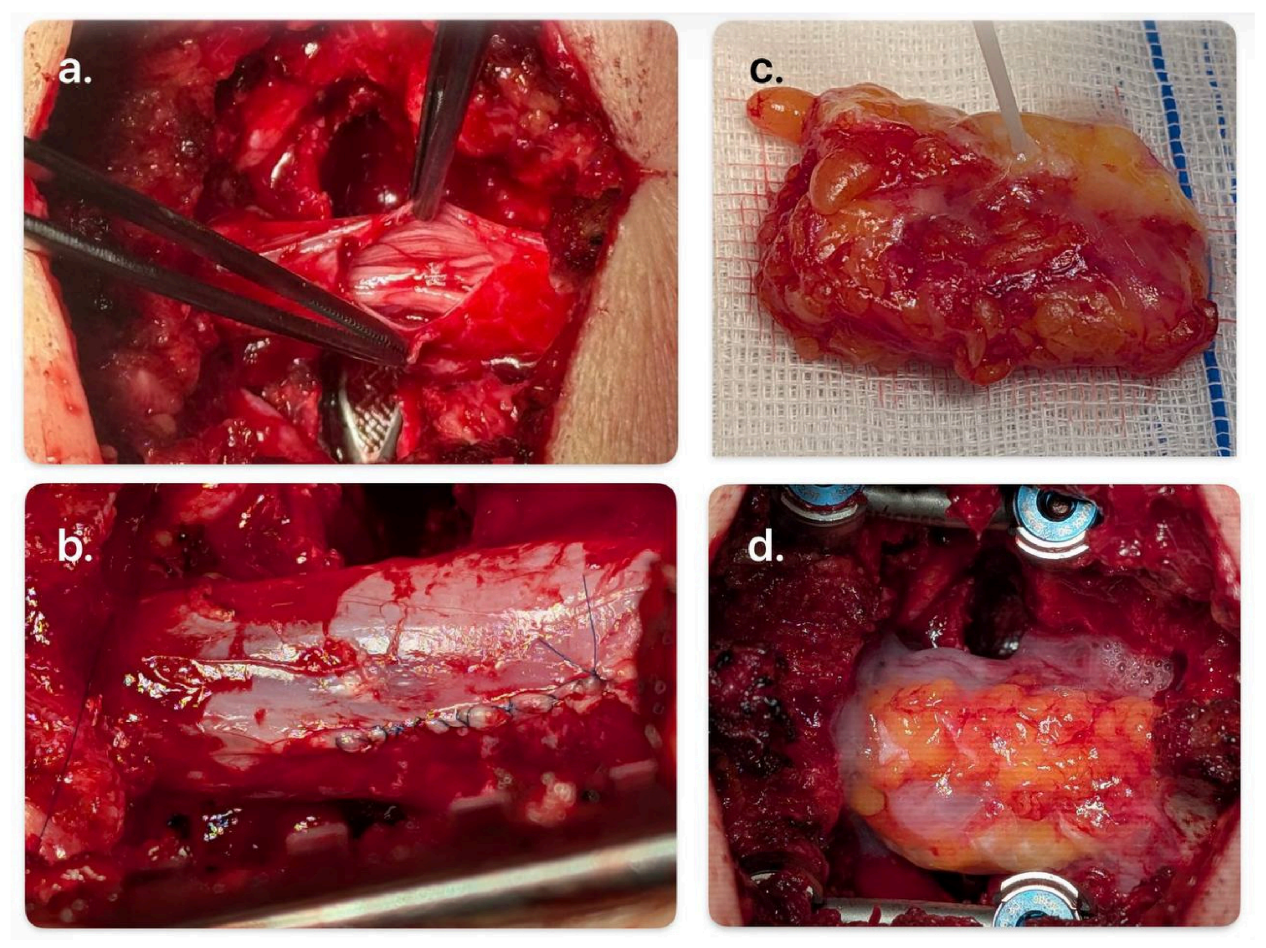
Intraoperative stages: (**a**) identification of dural tear; (**b**) microsurgical primary closure; (**c**) autologous graft preparation; (**d**) graft reinforcement of repaired dura.

After graft placement and drainage, thoracolumbar fascia was closed using interrupted Vicryl 2-0 sutures (Ethicon, Somerville, NJ, USA), the subcutaneous layer with Vicryl 0, and skin with interrupted vertical mattress Mersilene 2-0 sutures (Ethicon, Somerville, NJ, USA). These elements reflect the reported institutional technique, not a separately evaluated intervention. General principles of surgical wound closure have been described elsewhere [15]. Figure 2 documents the intraoperative repair stages.

### 2.3. Perioperative Management

Within the institutional 4D pathway, ceftriaxone 2 g was administered preoperatively and continued daily for five days; teicoplanin 400 mg was also administered preoperatively and during the drainage period. These regimens reflect the institutional practice of the reported period rather than a recommendation for prolonged routine antibiotic prophylaxis. Patients were maintained supine for five days and subsequently mobilized with a reinforced lumbar orthosis. The passive drain was generally maintained for at least seven days and removed after mobilization once output fell below 50 mL per 24 h. These postoperative measures were part of the reported treatment pathway and were not evaluated as independent interventions.

All patients developing postoperative wound dehiscence underwent prompt surgical revision under sterile conditions. Deep intraoperative cultures were systematically obtained from paraspinal musculature, fascial planes, and subcutaneous tissue. Antibiotic therapy was adjusted according to culture and sensitivity results when positive, or maintained empirically in culture-negative cases. All patients were followed with serial clinical assessments and inflammatory markers (C-reactive protein, erythrocyte sedimentation rate, and leukocyte count) until complete wound healing.

### 2.4. Outcomes and Statistical Methods

Patient characteristics, operative features, and postoperative outcomes were reported as counts and percentages or means ± standard deviations. Reported outcomes comprised persistent CSF leakage, symptomatic pseudomeningocele, CSF fistula, wound dehiscence, postural headache, meningitis, all-cause reoperation, length of stay, and drain duration and volume. Individual adverse events could coexist within the same patient; therefore, categories were not summed into a composite failure endpoint. Reoperation was retained as an all-cause descriptive endpoint because indications for every procedure were not delineated in the available aggregate report. No comparison with historical controls, statistical efficacy testing, or causal treatment-effect estimation was undertaken.

## 3. Results

### 3.1. Patient and Surgical Characteristics

The series comprised 112 patients receiving 4D repair. Mean age was 64.1 ± 9.2 years, and 60 patients (53.6%) were male. Mean body mass index was 25.1 ± 2.5 kg/m^2^; active smoking was recorded in 75 patients (67.0%) and diabetes mellitus in 44 (39.3%). Instrumented fusion was performed in 101 patients (90.2%), while 11 (9.8%) underwent decompression alone. Mean operative time was 190 ± 110 min and mean estimated blood loss 397 ± 144 mL (Table 1). Primary-versus-revision counts were not analyzed because a consistent denominator for this breakdown was unavailable from the aggregated records.

### 3.2. Dural Tear Characteristics

Small tears (<5 mm) occurred in 54 patients (48.2%), medium tears (5–10 mm) in 48 (42.9%), and large tears (>10 mm) in 10 (8.9%). The most common location was dorsal (90/112; 80.4%). The tear was recognized intraoperatively in 108 patients (96.4%). Available morphology data described 70 linear, 19 irregular or ragged, and 18 pinpoint tears (107 categorized lesions); morphology was not specified in the available summary for the remaining five patients (Table 2).

### 3.3. Postoperative Outcomes

Persistent CSF leakage was reported in 3 patients (2.7%), wound CSF fistula in 1 (0.9%), and wound dehiscence in 2 (1.8%). No symptomatic pseudomeningocele or postoperative meningitis was documented. Nine patients (8.0%) underwent at least one reoperation for any indication; two patients (1.8%) underwent revision for wound complications. Postural headache was recorded in 51 patients (45.5%). Mean hospital stay was 9.1 ± 3.8 days. Passive drainage lasted a mean of 7.5 ± 2.3 days, with a mean cumulative output of 1169 ± 347 mL during postoperative days 1–7 (Table 3). The two wound-dehiscence events are reported as wound complications, without presuming microbiologically confirmed infection.

## 4. Discussion

This series describes the surgical sequence and observed postoperative course of 112 patients managed with a standardized multimodal approach to incidental durotomy. The principal contribution is practical: an illustrated and potentially reproducible technique combining meticulous primary closure with sealant, autologous soft-tissue reinforcement, and passive drainage. The descriptive observations include three recorded persistent CSF leaks, two wound dehiscences, and nine reoperations. These figures document the experience at one institution but cannot demonstrate that the bundle is more effective than another repair strategy.

The published literature shows that incidental dural tears occur across a broad spectrum of spinal operations and may affect perioperative morbidity [1,2,3,4,5,13,16]. Systematic reviews of dural repair techniques emphasize the heterogeneity of surgical materials, defect morphology, and definitions of failure [9,10]. Direct primary closure remains foundational whenever feasible, while adjuncts such as grafts and sealants may be selected according to defect geometry and tissue quality. The present series contributes a detailed technical illustration rather than a controlled effectiveness estimate.

The first two 4D components aim to provide a watertight mechanical closure using double continuous suturing supplemented with fibrin sealant. The free autologous dermal-fat graft provides an additional tissue covering. Prior technical reports have described fat grafting for spinal CSF leakage prevention and repair [12], while systematic reviews have evaluated sutures, sealants, and graft combinations across heterogeneous dural defects [9,10]. An in vitro study has assessed the watertight performance of dural sutures and patches [17], while biomaterial research has explored bacterial cellulose-based CSF barrier substitutes [18]. Neither line of experimental evidence establishes clinical effectiveness of the present 4D protocol. Since all patients in this series received the bundled approach, the relative contribution of suturing, sealant, and grafting cannot be isolated.

The fourth component uses gravity-dependent passive subfascial drainage. Wilson et al. evaluated a subfascial epidural drainage protocol after thoracic and lumbar durotomy, and Bauerle et al. described passive bile-bag drainage as another management strategy [19,20]. These reports provide clinical context but differ in population and perioperative details; they cannot establish direct equivalence or superiority to the present technique. In the 4D pathway, drains were maintained for approximately one week and patients were kept supine for five days. These durations represent historical institutional management rather than established optimal practice. A systematic review and meta-analysis of early ambulation versus prolonged bed rest highlighted the importance of reassessing routine immobilization [21]. Likewise, the absence of meningitis in this series cannot alone establish drainage safety, particularly with concomitant extended antibiotic administration.

Comparison with published reports should therefore remain qualitative. Systematic reviews by Alshameeri et al. and Choi et al. emphasize variability in repair combinations and outcome definitions [9,10]. The institutional experience and literature review by Albayar et al. also illustrate the central role of primary closure and the use of adjuncts tailored to the defect [13]. The present series documented persistent CSF leakage in 3/112 patients (2.7%), wound dehiscence in 2/112 (1.8%), and all-cause reoperation in 9/112 (8.0%). These descriptive estimates must not be ranked against outcomes from other cohorts without accounting for differences in case mix, follow-up, definitions, and reoperation indications.

Limitations include the retrospective, single-center design, lack of a contemporaneous comparator, and the inability to determine the independent effects of individual protocol components. Detailed indications for all nine reoperations and microbiological confirmation of surgical-site infection were not available as separately adjudicated outcomes. In addition, five tear morphologies were not specified in the summary records; longer-term follow-up denominators, uniform imaging surveillance, validated patient-reported outcomes, and posturographic measurements were not available for this descriptive analysis. Accordingly, this study documents a surgical approach and its observed clinical course rather than demonstrating relative effectiveness. Patient-oriented aesthetic assessments following lumbar surgery [22] and quantitative posturographic assessment methods [23] have been reported in other settings, but were not examined in this series.

Future studies should prospectively define standardized CSF-related outcomes, adjudicate indications for reoperation, capture wound cultures separately from mechanical dehiscence, report imaging and patient-reported results, and compare alternative repair and postoperative-management strategies under contemporary conditions. Such work could help identify which elements of a multimodal technique add meaningful benefit.

## 5. Conclusions

The 4D protocol combines double-layer dural suturing, sealant reinforcement, autologous dermal-fat grafting, and passive subfascial drainage in a clearly documented operative sequence. In this single-center retrospective series of 112 patients, selected CSF-related complications and reoperations were reported with descriptive frequencies. These data support reporting the technique as a practical institutional experience, but neither establish comparative efficacy nor justify routine prolonged drainage, bed rest, or extended antibiotic prophylaxis. Prospective independent validation is warranted.

## Figures and Tables

**Table 1:** Patient and operative characteristics in the 4D series (*n* = 112).

Variable	4D Technique (*n* = 112)
Mean age, years ± SD	64.1 ± 9.2
Male sex, *n* (%)	60 (53.6)
Female sex, *n* (%)	52 (46.4)
Mean BMI, kg/m^2^ ± SD	25.1 ± 2.5
Active smoker, *n* (%)	75 (67.0)
Diabetes mellitus, *n* (%)	44 (39.3)
Lumbar surgery, *n* (%)	111 (99.1)
Thoracic surgery, *n* (%)	1 (0.9)
Decompression alone, *n* (%)	11 (9.8)
Instrumented fusion, *n* (%)	101 (90.2)
Mean treated levels ± SD	1.9 ± 1.1
Mean operative time, min ± SD	190 ± 110
Mean estimated blood loss, mL ± SD	397 ± 144

**Table 2:** Reported intraoperative characteristics of dural tears in the 4D series (*n* = 112).

Variable	4D Technique (*n* = 112)
TEAR SIZE	
Small (<5 mm), *n* (%)	54 (48.2)
Medium (5–10 mm), *n* (%)	48 (42.9)
Large (>10 mm), *n* (%)	10 (8.9)
TEAR MORPHOLOGY	
Linear, *n* (%)	70 (62.5)
Irregular/ragged, *n* (%)	19 (17.0)
Morphology not specified in aggregate record, *n* (%)	5 (4.5)
Pinpoint, *n* (%)	18 (16.1)
TEAR LOCATION	
Dorsal, *n* (%)	90 (80.4)
Lateral, *n* (%)	15 (13.4)
Ventral, *n* (%)	7 (6.3)
Intraoperative recognition, *n* (%)	108 (96.4)

**Table 3:** Reported clinical outcomes following 4D repair (*n* = 112). Events are not mutually exclusive.

Outcome Variable	4D Technique (*n* = 112)
Persistent CSF leakage, *n* (%)	3 (2.7)
Symptomatic pseudomeningocele, *n* (%)	0 (0.0)
Wound CSF fistula, *n* (%)	1 (0.9)
Wound dehiscence, *n* (%)	2 (1.8)
Postural headache, *n* (%)	51 (45.5)
Postoperative meningitis, *n* (%)	0 (0.0)
Revision surgery for wound complications, *n* (%)	2 (1.8)
Any reoperation, *n* (%)	9 (8.0)
Mean hospital stay, days ± SD	9.1 ± 3.8
Mean drainage duration, days ± SD	7.5 ± 2.3
Mean cumulative drainage volume, POD1–7, mL ± SD	1169 ± 347

## Data Availability

The original contributions presented in this study are included in the article. Further inquiries can be directed to the corresponding author.
